# Lateral Lumbar Interbody Fusion at the L5-S1 Vertebral Level: A Unique Anatomical Case Report

**DOI:** 10.7759/cureus.22096

**Published:** 2022-02-10

**Authors:** Michael J Spitnale, Zachary T Thier, Gregory Grabowski

**Affiliations:** 1 Orthopedic Surgery, Prisma Health–Midlands/University of South Carolina, Columbia, USA; 2 Medical Education, Lincoln Memorial University–DeBusk College of Osteopathic Medicine, Knoxville, USA

**Keywords:** far lateral interbody fusion, lumbosacral spine, degenerative spine disease, mis, transitional anatomy, spine deformity

## Abstract

A 57-year-old female presented with L4-L5 and L5-S1 mobile spondylolisthesis and associated stenosis with radiculopathy who failed conservative treatment. This patient underwent lateral lumbar interbody fusion (LLIF) of L4-L5 and L5-S1, and posterior spinal fusion (PSF) with instrumentation.

LLIF is a minimally invasive procedure to treat degenerative diseases of the lumbar spine. LLIF at the L5-S1 vertebral level is a relative contraindication secondary to increased risk of injury to the lumbar plexus and access issues at this level during the approach. With the help of imaging, careful preoperative planning can make this a feasible procedure in select patients.

## Introduction

Lateral lumbar interbody fusion (LLIF) is a relatively new and minimally invasive technique for interbody fusion and is also referred to as direct lateral interbody fusion [1-3]. This surgical procedure falls in the family of lumbar interbody fusion procedures along with posterior lumbar interbody fusion (PLIF), transforaminal lumbar interbody fusion (TLIF), anterior lumbar interbody fusion (ALIF), minimally invasive transforaminal lumbar interbody fusion (MI-TLIF), and oblique lumbar interbody fusion (OLIF). LLIF uses a flank incision and direct lateral access to the intervertebral disc via the retroperitoneal space [2]. Due to the minimally invasive properties, LLIF has been indicated for degenerative spinal diseases such as spinal stenosis, spondylolisthesis, scoliosis, and disc degenerative diseases [2]. It has shown comparative advantages over the other procedures of its kind, as it allows for a wider interbody cage to span the lateral borders of the apophyseal ring bilaterally and preserve the annular and ligamentous structures such as the anterior longitudinal ligament [2].

While it has shown success for degenerative diseases of the spine, there are relative anatomical contraindications that make this a technically difficult and risky procedure to complete. At the L5-S1 level, the LLIF procedure approach becomes less straightforward due to the anatomical position of the iliac crest. The top of the iliac crest blocks straight access to the vertebral column at the L5-S1 level because the surgeon would have to traverse over the top of the iliac crest and down to the disc level at an inferiorly directed angle. Another relative anatomical contraindication is the position of the psoas muscle. As the vertebral column descends, the psoas muscle will move anteriorly, which makes the lumbosacral plexus more vulnerable because of how it courses in the muscle [4]. The position of the great vessels (abdominal aorta, inferior vena cava, and common iliac arteries and veins) over the vertebral bodies is also a concern during this approach. The great vessels can course over the anterolateral portions of the vertebral bodies, presenting a risk of injury during the approach to the lateral portion of the vertebral bodies.

It has been globally considered to not perform an LLIF at the L5-S1 level, but we report a case where multiple anatomical differences of this patient allow for the use of LLIF at the L5-S1. With the use of radiographic and other advanced imaging, the LLIF procedure may be performed if the correct anatomical considerations have been individually applied to each case.

Statement of informed consent

The patient was made aware that her data would be submitted for publication, and the patient agreed.

## Case presentation

A 57-year-old Hispanic female presented with numbness and tingling in the toes on her right side and low back pain localized to the left side. She presented with bilateral 5/5 muscle strength in the extensor hallucis longus, tibialis anterior, gastrocnemius, soleus, peroneals, and quadriceps. The pain had kept her from completing daily activities such as rising from the table, and she reported feeling crippled every time she would attempt to exercise. She failed conservative therapy with nonsteroidal anti-inflammatory medication, physical therapy, and epidural steroid injections.

She was diagnosed with mild degenerative scoliosis most notable at the L4-L5 and L5-S1 levels, spinal stenosis, and grade I anterior spondylolisthesis at the L5-S1 level. The patient was also noted to have a partially lumbarized S1 vertebra. Although this is a transitional vertebra that could have represented an L4-L5 disc space in a normal anatomical person, our patient still had five lumbar, 12 thoracic, and seven cervical vertebrae above this S1 level. Therefore, this was still the patient’s true L5-S1 disc space, even with the partially lumbarized S1 vertebra. Surgical treatment was discussed as a possibility since the patient failed conservative therapy, and the potential complications and risks were explained.

**Figure 1:**
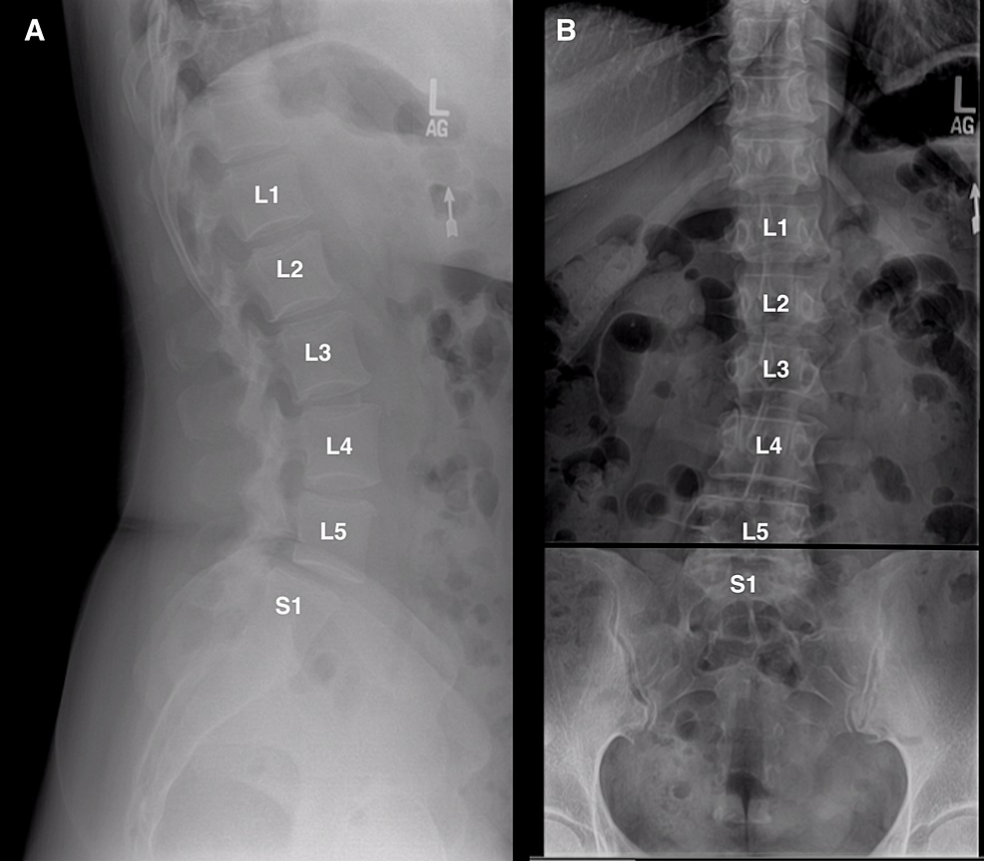
Lateral (A) and anterior–posterior (AP) (B) radiographs showing where the iliac crest is sitting right at the L5 vertebral column (black line), along with the lumbarized S1.

**Figure 2:**
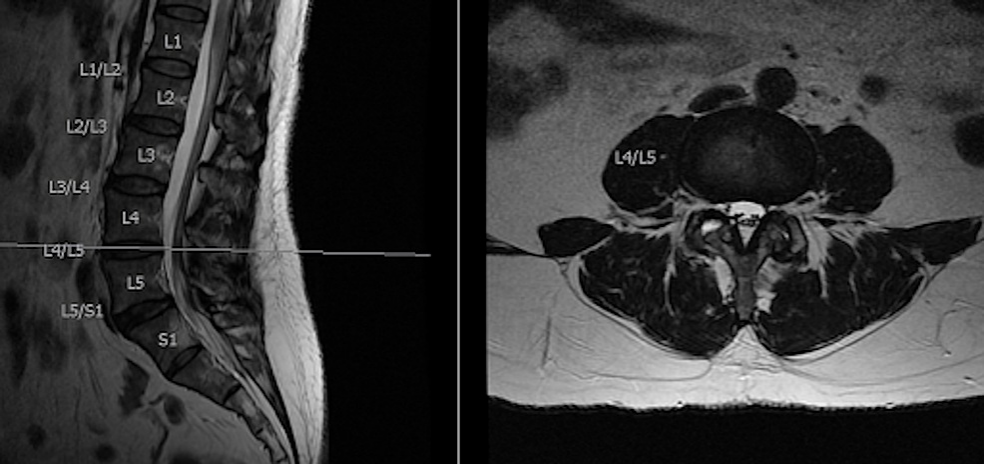
Sagittal and axial cuts at the L4-L5 interspace.

**Figure 3:**
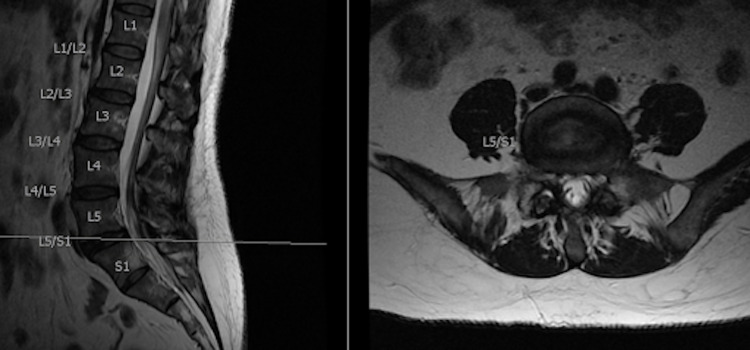
Sagittal and axial cuts at the L5-S1 interspace.

With the use of radiographic and magnetic resonance imaging, anatomical differences were observed, which allowed for the use of LLIF at the L5-S1 level. The patient had three different anatomical considerations that made the LLIF at the L5-S1 level possible. First, the patient’s iliac crest was positioned at the inferior aspect of the L5 vertebral body, while the average top of the iliac crest is between the L4 vertebral body down to the L4-L5 disc space (Figure 1). Second, the patient’s psoas muscle is more posteriorly oriented at the L5-S1 disc space, compared to an average person where the psoas is more anteriorly positioned at this disc space. This is an important anatomical consideration for avoiding the lumbar plexus located within the psoas because, by the time the psoas courses down to the L5-S1 disc space, the lumbar plexus is at a much higher risk of injury during the surgical dissection because of its more anterior orientation (Figures 2-3). The last important anatomical consideration seen on imaging was the location of the great vessels relative to the vertebral bodies at the L5-S1 level. The patient’s great vessels are more anterior to the vertebral bodies, making these structures at a lower risk of injury during surgical dissection (Figures 2-3). After these anatomical considerations were observed, the patient was informed that the LLIF procedure, combined with percutaneous screw fixation, could be done due to her unique anatomy. She was also informed that intraoperative neuromonitoring would be utilized to reduce the risk of any neurological injury during the approach and make sure that this particular surgery at this unique level would still be as successful as possible. The surgery was a success both perioperatively and postoperatively, and the patient began postoperative recovery.

**Figure 4:**
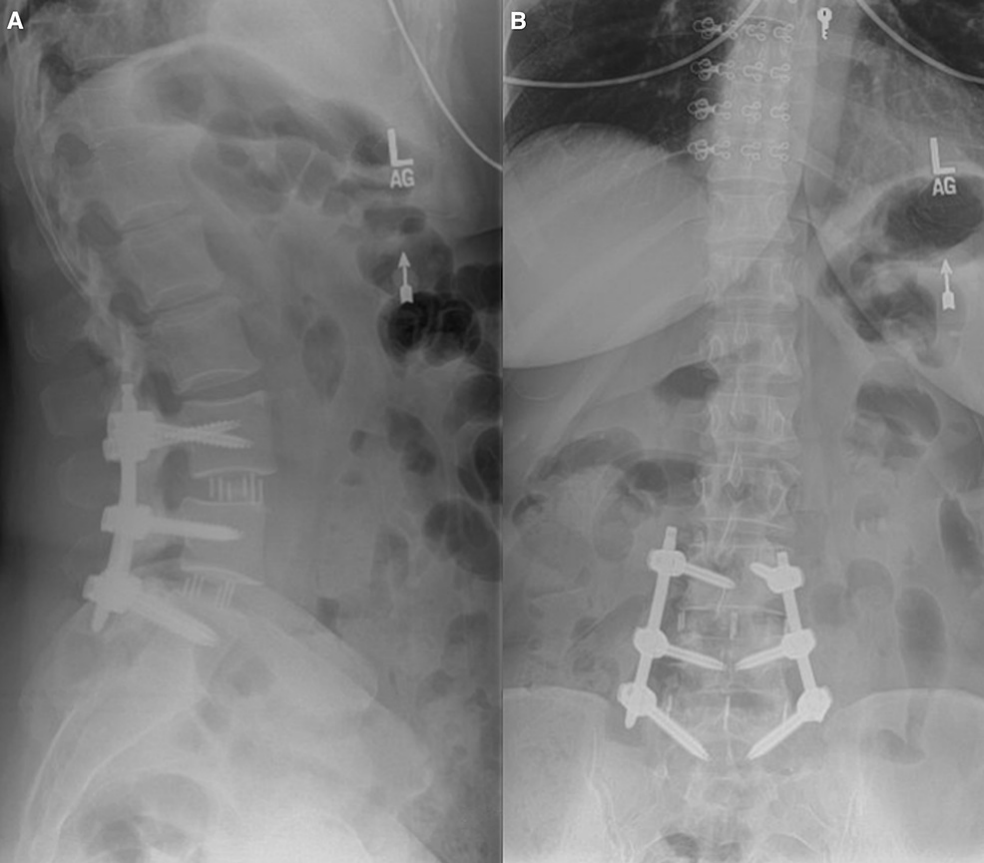
Lateral (A) and AP (B) radiograph of the lumbar spine of the patient at the six-week postoperative period showing improved alignment.

At the six-week postoperative appointment, the patient improved exceptionally well. The patient reported that she had minimal pain in the low back with resolution of the radicular symptoms but had no complaints otherwise. Lateral and anterior-posterior (AP) views of the fusion were obtained, which showed improvement of vertebral alignment with no signs of hardware loosening or breakage (Figure 4). Activities were increased, and more imaging was acquired at subsequent follow-up appointments.

**Figure 5:**
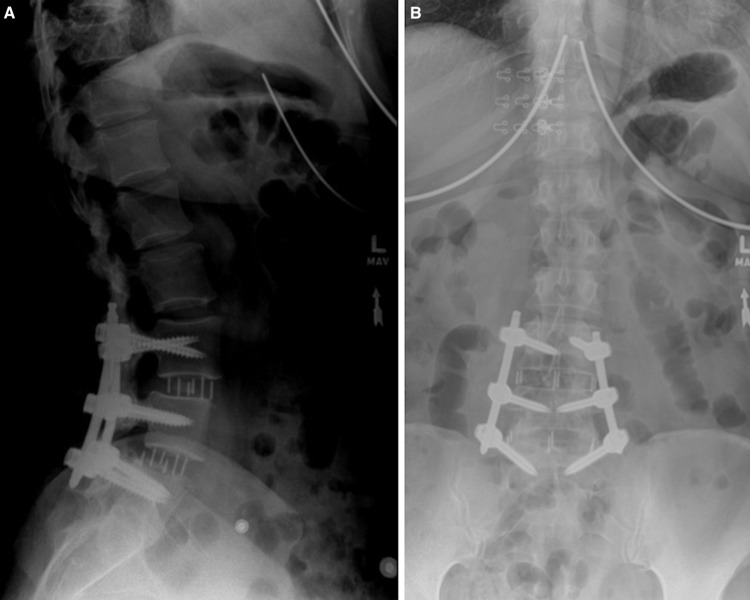
Lateral (A) and AP (B) radiograph of the lumbar spine at the six-month postoperative period.

At the patient’s six-month follow-up, she reported that she felt 100% better than her baseline rating preoperatively. She reported that her lower back and extremities were satisfactory, and she was participating in all activities, except for heavy lifting. Follow-up radiographic imaging of the lumbar spine showed no change from the previous follow-up (Figure 5). At her 20-month follow-up, she stated having no pain or limitations in her daily activities and only noticed pain when she was lifting heavy objects. Our patient completed the RAND Health Care 36-Item Health Survey (RAND) and the Oswestry Low Back Pain Scale (OLBPS) pre- and postoperatively. In the OLBPS, our patient recorded a 70% total disability preoperatively, 30% total disability in the postoperative period, and 2% total disability score at 20-month follow-up. The total RAND score preoperatively was 38, 6-month postoperatively was 82, and at 20-month follow-up was 92.

## Discussion

LLIF is a procedure used to correct spinal degenerative diseases. A retrospective study showed that the LLIF approach improved the Oswestry Disability Index, Roland-Morris Disability Index, and EQ-5D VAS scores significantly compared with their baseline scores, which tests functionality, physical disability due to low back pain, overall health status, and the intensity and frequency of symptoms [5]. While they were able to help these patients, there are still anatomical considerations that should be considered as the current literature does not support the use of LLIF at the L5-S1 level due to certain considerations. In normal anatomical considerations, the iliac crest blocks the lateral parallel entrance at the L5-S1 interspace because the iliac crest normally sits parallel to the L4-L5 interspace. If taking a more caudal and oblique approach due to the shape of a normal anatomical iliac crest, the neurovascular structures are more exposed to injury [6,7]. Figure 1 shows our patient’s AP/lateral radiographs with the iliac crest sitting parallel to the L5-S1 interspace, as opposed to the normal L4-L5 interspace.

In a study by Benglis et al. in 2009, it was found that within the psoas muscle from L1 to L5, the lumbar plexus has a natural posterior to anterior migration [8]. This is an important anatomical consideration for avoiding the lumbar plexus located within the psoas, because by the time the psoas courses down to the L5-S1 disc space, the lumbar plexus is at a much higher risk of injury during the surgical dissection because of its more anterior orientation. Our patient differed in this anatomy as the psoas muscle was more posterior at the L5-S1 level as seen in Figure 2. This could be explained by her lumbarized S1 vertebra, but given that she had all five lumbar, 12 thoracic, and seven cervical vertebrae above this S1 level, we felt that it acted more like a true L5-S1 interspace. The last important anatomical consideration is the locations of the great vessels relative to the vertebral bodies at the L5-S1 level. The more inferior the lumbar spine courses, the more laterally the great vessels span, which increases the risk of injury for LLIF [6,7].

Due to our patient’s unique anatomical considerations, we were able to conduct the LLIF procedure, in the midst of the considerations, through careful preoperative planning with the help of radiographic and magnetic resonance imaging. It is these anatomical considerations that allowed us to perform an LLIF on a patient at the L5-S1 level. This shows that this procedure can be done at the L5-S1 interspace with the use of quality imaging and appropriate interpretation, intraoperative neuromonitoring, and understanding the potential impact of the transitional vertebra and its surrounding neurovascular structures to ensure the anatomical considerations match the desired criteria.

## Conclusions

LLIF is a minimally invasive procedure to treat degenerative diseases of the lumbar spine. LLIF at the L5-S1 vertebral level is a relative contraindication secondary to increased risk of injury to the lumbar plexus and access issues at this level during the approach. Due to the unique anatomy of this patient, we were able to safely and successfully perform the LLIF procedure despite the relative contraindications. With the help of imaging, careful preoperative planning can make this a feasible procedure in select patients.
